# HIRA directly targets the enhancers of selected cardiac transcription factors during in vitro differentiation of mouse embryonic stem cells

**DOI:** 10.1007/s11033-018-4247-z

**Published:** 2018-07-20

**Authors:** Rasha Noureldin M. Saleh, Daniel Dilg, Abla A. Abou Zeid, Doaa I. Hashad, Peter J. Scambler, Ariane L. A. Chapgier

**Affiliations:** 1 Developmental Biology of Birth Defects Section, Institute of Child Health, University College London, London, UK; 2 Clinical Pathology Department, Faculty of Medicine, Alexandria University, Alexandria, Egypt

**Keywords:** HIRA, H3.3, Heart, Cardiomyocytes, Embryonic stem cells, Differentiation, Enhancers

## Abstract

HIRA is a histone chaperone known to modulate gene expression through the deposition of H3.3. Conditional knockout of *Hira* in embryonic mouse hearts leads to cardiac septal defects. Loss of function mutation in *HIRA*, together with other chromatin modifiers, was found in patients with congenital heart diseases. However, the effects of HIRA on gene expression at earlier stages of cardiogenic mesoderm differentiation have not yet been studied. Differentiation of mouse embryonic stem cells (mESCs) towards cardiomyocytes mimics some of these early events and is an accepted model of these early stages. We performed RNA-Seq and H3.3-HA ChIP-seq on both WT and *Hira*-null mESCs and early cardiomyocyte progenitors of both genotypes. Analysis of RNA-seq data showed differential down regulation of cardiovascular development-related genes in *Hira*-null cardiomyocytes compared to WT cardiomyocytes. We found HIRA-dependent H3.3 deposition at these genes. In particular, we observed that HIRA influenced directly the expression of the transcription factors *Gata6, Meis1* and *Tbx2*, essential for cardiac septation, through H3.3 deposition. We therefore identified new direct targets of HIRA during cardiac differentiation.

## Introduction

Heart development is a complex process involving cardiogenic mesodermal differentiation into endocardium and myocardium. The myocardium is generated sequentially from cardiac precursors, primitive cardiomyocytes and definitive cardiomyocytes [1]. It requires a tightly controlled temporal gene expression pattern, which if disturbed can lead to the development of congenital heart diseases [2]. For example, mutations in *Gata4* or *Tbx2*, important cardiac transcription factors, are associated with endocardial cushion and cardiac septal defects [3, 4]. Chromatin modifications and histone variants are essential for lineage commitment and cell fate [5, 6]. A distinct chromatin pattern specific for each stage of stem cell differentiation into cardiomyocytes was reported [7]. The histone variant H3.3 plays a role in early embryonic development [8] and lineage commitment of stem cells [9–11]. HIRA is required for genome wide enrichment of the histone variant H3.3 at active and bivalent genes in mouse embryonic stem cells (mESCs) [11].

HIRA is part of one of the two complexes that deposits H3.3 in a replication independent manner [12, 13]. The HIRA complex includes UBN1, CABIN1 and ASF1 [14]. It deposits H3.3 at the gene bodies of developmentally regulated genes [11], highly active genes [10] and some active enhancers and regulatory elements [15]. It has also been shown that there is HIRA-independent H3.3 enrichment at certain transcription factor binding sites (TFBS) and at telomeres in undifferentiated mESCs in a ATRX/DAXX dependent manner [10].

Absence of HIRA in mice [16] and Xenopus laevis [17] leads to gastrulation defects and embryonic lethality. Attenuation of *Hira* expression in chick cardiac neural crest results in common arterial trunk, suggesting a role for HIRA in outflow tract (OFT) septation [18]. *Hira*-null mouse embryos showed abnormal heart looping and pericardial oedema at embryonic day (E)10.5 [16]. Recently, *HIRA* was found to be one of 46 chromatin modifiers that had a significant loss of function mutation (*p* value = 0.03) in a large congenital heart disease cohort study [19]. We previously published that conditional knockout of *Hira* in the cardiogenic mesoderm resulted in embryonic lethality with atrial and ventricular septal defects at E15.5. Following genome-wide analysis of the transcriptome of these conditional knockout mouse hearts at E12.5, we identified a dysregulation of certain cardiac genes such as *Tnni2, Tnnt3* and *Epha3* [20]. We showed HIRA-dependent H3.3 deposition at the enhancer *Tte* that regulates the expression of *Tnni2* and *Tnnt3* during cardiac development.

However, the role of HIRA at the early stages of cardiogenic mesoderm differentiation has not been studied. Differentiation of mESCs towards cardiomyocytes mimics the early stages of cardiogenic mesodermal differentiation [7, 21]. Here, we used in vitro mESC differentiation to study the role of HIRA during this process and focused on day 15, when the cardiogenic mesoderm genes are expressed [22]. High-throughput RNA sequencing (RNA-seq) and H3.3-HA chromatin immunoprecipitation followed by massive parallel sequencing (ChIP-seq) were then performed on both WT and *Hira*-null undifferentiated and mESC-derived cardiomyocytes at day 15 of differentiation. We report here that HIRA is required for the expression of cardiac transcription factors involved in septation during cardiac development. These transcription factors displayed diminished H3.3 enrichments in their gene bodies or near their Transcription Start Site (TSS) in the absence of HIRA. This reflects the requirement for HIRA in the expression of important cardiac transcription factors through H3.3 deposition.

## Methods

### Cell culture and differentiation

H3.3-HA tagged wild type (W9.5) and *Hira*-null (Clone 104) mESCs have been cultured and maintained as described previously [22]. These cells were maintained in an undifferentiated state at 37 °C and 5% CO_2_ on 0.1% gelatin coated flasks in Knockout™ D-MEM (GIBCO, 10829), supplemented with 15% ES-FCS (Millipore ES-009B), 1× Glutamax (GIBCO 35050-038), 1× Penicillin/Streptomycin (GIBCO 15140), 1× MEM NEAA (GIBCO 11140-035), 0.1 mM 2-β-mercaptoethanol (SIGMA M-7522) and 10^3^ Units/ml LIF (Millipore, ESG-1106).

Differentiation was adapted from the previously described hanging drop method, with some modifications [22, 23]. Briefly, cells were cultured in a medium made of DMEM (GIBCO 61965-026), with 15% ES-FCS (Millipore ES-009B), 1× Penicillin/Streptomycin (GIBCO 15140), 1× MEM NEAA (GIBCO 11140-035) and 0.1 mM β-mercaptoethanol (SIGMA M-7522). Embryoid bodies (EBs) were formed by hanging the cells in the form of drops on the lids of petri dishes at a concentration of 25 cell/µl. After 2 days, these EBs were dropped into non-TC treated petri dishes and were grown in suspension culture for another 2 days. At day 4, the EBs were plated in 0.1% gelatin coated tissue culture dishes and the medium was changed every day till day 15.

### RNA extraction and sequencing

RNA was extracted from two different biological samples of both WT and *Hira*-null cells at day 0 and day 15 using TRIzol reagent (Life technologies 15596-018) following the manufacturer’s instructions. The quality and quantity of RNA were determined using Nanodrop spectrophotometer ND-1000 (Lab tech) and using High Sensitivity RNA screenTape®, samples with RIN of seven or more were used for subsequent library preparation. Library preparation was done using KAPA stranded mRNA-seq kit (KAPABIOSYSTEMS KK8421) and KAPA mRNA capture kit (KAPABIOSYSTEMS KK8441). RNA sequencing was performed by Illumina NextSeq 500, with the production of paired-end reads. Reads were aligned to mouse genome mm9 using bowtie [24] and differential expression was processed using Deseq package version 1.6.3. The produced gene lists were sorted using an adjusted *p* value ≤ 0.05 and an absolute fold change of ± 2. Gene Ontology analysis was done using DAVID bioinformatics functional annotation tool [25]. Enrichment analysis was performed using gene set enrichment analysis (GSEA) software and the candidate genes were selected using the GSEA lead Edge tool [26]. Functional enrichment was visualized using Cytoscape to produce enrichment maps [27].

### Reverse transcription and quantitative real time PCR

The High-Capacity RNA-to-cDNA™ Kit (Thermo fisher 4387406) was used to obtain cDNA and was used according to the manufacturer’s instructions. Primers for qRT-PCR were designed using the primer-blast tool of NCBI. The amplicon size was set between 70 and 200 bp (bp). Quantitative real time PCR (qRT-PCR) was performed on the CFX96 Touch™ Real-Time PCR Detection System using SYBR green (BIO-RAD 1708882) on three biological replicates and analysis was done using the standard curve method. Results were normalized to *Gapdh*.

### Native ChIP followed by qPCR

Native ChIP was performed on 1 × 10^7^ cells as previously described [10, 11]. Briefly, the cell pellets were lysed and chromatin was digested using MNase (Sigma N5386) at 37 °C with an ideal fragmentation between 200 and 1000 bp. The reaction was stopped using 5 mM EDTA. HA antibody was collected with dynabeads (Invitrogen, 112.03D, 112.01D) from 12AC5 hybridoma supernatants and then incubated with the lysates overnight at 4 °C. After several washes, the samples were treated with proteinase K and RNAse. DNA was purified using the PCR-purification Qiagen kit (28104). Quantitative real time PCR following ChIP (q-ChIP) or ChIP-seq were performed.

Primers for validation of ChIP-seq by q-ChIP were designed as follow; H3.3 significant peaks were uploaded in Integrative Genome browser (IGV). H3.3 enriched and depleted regions were identified. The corresponding nucleotide sequences were then extracted and used for primer design using NCBI primer design tool (https://www.ncbi.nlm.nih.gov/tools/primer-blast/).

### ChIPseq and data analysis

The eluted DNA from both Input and ChIP was processed through library preparation using the NEB DNA Ultra kit and selecting fragment sizes of around 200 bp. Samples were sequenced using Illumina NextSeq 500, with the production of paired-end reads. Reads were aligned to mouse genome mm9 using BOWTIE 1.1.2 allowing no more than three mismatches [28]. Samtools 1.3.1 was used to generate BAM files, remove duplicates, sort and index [29]. MACS 1.4.2 was used for peak calling using the default parameters (*p* value ≤ 10^−5^) [30]. BEDTOOLS intersect with (–v) option was used to generate the HIRA-dependent H3.3 peaks, after excluding the peaks in the *Hira*-null sample [31]. Gene lists were generated within + 10 kb of the TSS, in the gene body and 200 bp dowstream TES using the PAPST (Peak Assignment and Profile Search Tool) tool [32]. Genome-wide distribution of the H3.3 ChIP-seq peaks was analyzed from both bed and wig files using the cis regulatory element annotation tools (CEAS) software [33]. The HIRA-dependent H3.3 peaks resulted from the subtraction of the *Hira*-null peaks from the WT peaks using bedtools.

## Results

### Cardiac markers are dysregulated in the absence of HIRA during early cardiomyocyte differentiation

**Fig. 1:**
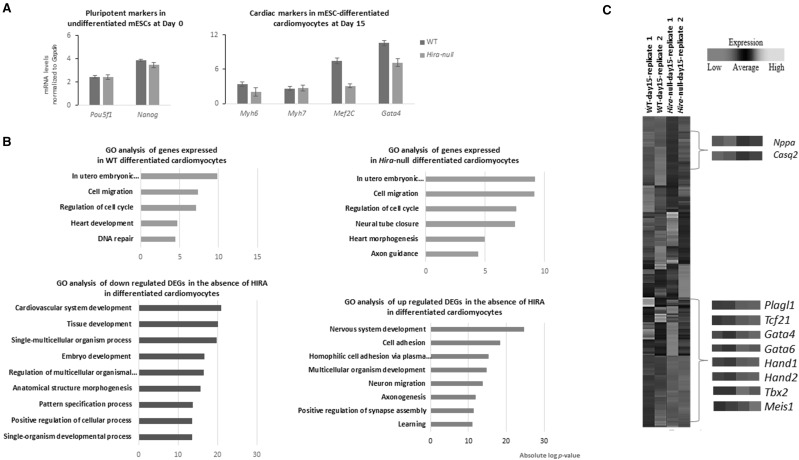
Cardiac genes expression is dysregulated in the absence of HIRA during the differentiation of mESCs into cardiomyocytes. **a** qRT-PCR on WT and *Hira*-null undifferentiated mESCs (Day 0) and differentiated cardiomyocytes (Day 15) displaying an upregulation of *Nanog* and *Pou5f1*, pluripotency markers, at Day 0. On the contrary, the cardiac specific markers *Myh6, Myh7, Mef2c and Gata4* are upregulated in mESCs-differentiated cardiomyocytes at Day 15. This experiment is representative of three biological experiments. The error bars represent variation between technical triplicates **b** Significantly enriched GO terms (biological process) of expressed genes (orange) in WT and *Hira*-null differentiated cardiomyocytes (adjusted *p* value ≤ 0.05 and FDR ≤ 0.1) and of down regulated (blue) or up regulated (red) DEGs in the absence of HIRA (adjusted *p* value ≤ 0.05 and FDR ≤ 0.1) in our RNA-seq analysis at day 15 of differentiation. **c** Heatmaps displaying the DEG levels in each of the cells at day 15 of differentiation. Genes related to cardiac development are magnified. Two biological replicates from distinct experiments are represented. (Color figure online)

WT and *Hira-*null mESCs were differentiated towards a cardiac mesodermal lineage using an adapted hanging drop protocol to increase the cardiomyocyte yield [22, 23]. Beating foci were present from day 8 of differentiation in both WT and *Hira-*null cells. These foci increased gradually in size during differentiation until reaching 60–70% confluence at day 15. These beating areas arose concomitantly with the expression of the cardiac transcription factor *Nkx2.5* at day 8 [20]. We confirmed the expression of *Gata4, Mef2c, Myh6* and *Myh7*, as cardiac specific marker at day 15 in both WT and *Hira*-null cells, and the expression of the pluripotency markers *Nanog* and *Pou5f1* at day 0, by qRT-PCR (Fig. 1a). We then sequenced the total RNA in undifferentiated and in differentiated WT and *Hira*-null cells at day 15. We compared the expressed genes observed in our experiment at day 15 with the dataset previously published by Wamstad et al. at the cardiomyocyte (CM) stage. We found that 68.6% and 67.4% of the expressed genes in WT and *Hira*-null dataset respectively overlap with the reported CM stage datasets, indicating that loss of HIRA does not prevent general cardiogenic differentiation.

Gene ontology (GO) biological process analysis was performed on the genes expressed in WT and *Hira*-null differentiated cardiomyocytes. Genes related to heart development and morphogenesis were expressed in both cell types, indicating successful differentiation into cardiac lineage in both cells (Fig. 1b). However, GO analysis of the differentially expressed genes (DEGs) show that genes related to cardiovascular development were relatively depleted in *Hira*-null compared to WT mESCs-differentiated cardiomyocytes (*p* value = 4.1 × 10^−21^). On the contrary genes related to the nervous system development were upregulated in the absence of HIRA. We found 1680 DEGs of which 1641 encode known genes including 108 cardiac genes and 39 were non-coding RNAs (*p* value ≤ 0.05 and FC = ± 2) (Fig. 1c).

**Fig. 2:**
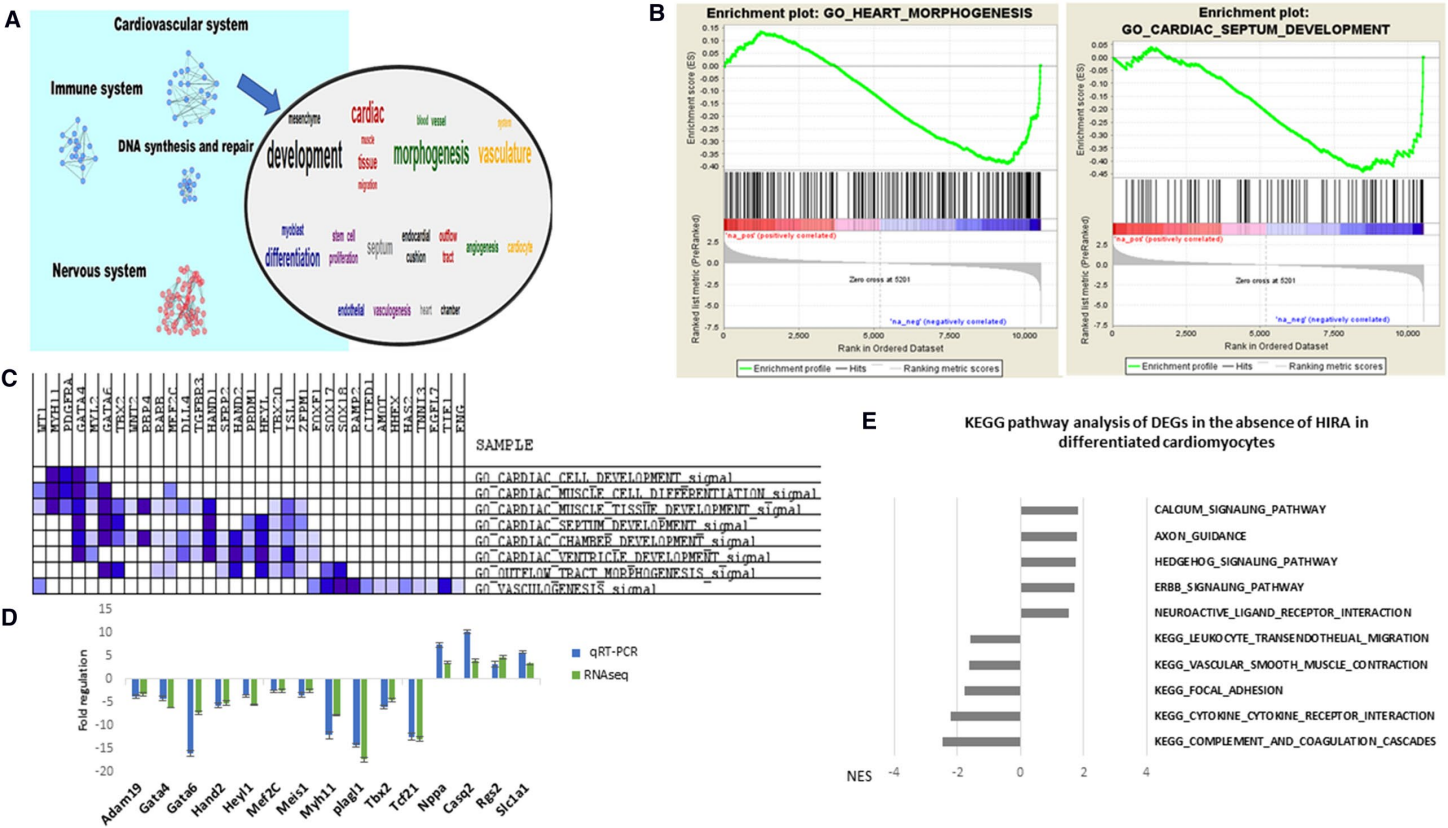
Genome-wide analysis of the effects of HIRA on transcription during the differentiation of mESCs into cardiomyocytes. **a** Enrichment map of gene sets and Word Cloud performed on the DEGs, with the highlight of the most frequent terms in the cardiovascular system cluster. Gene sets in blue are down regulated and the one in red are up regulated in the absence of HIRA. **b** Enrichment plots displaying the negative enrichment of GO Heart morphogenesis (NES = − 1.65, FDR = 0.08) and cardiac septum development (NES = − 1.67, FDR = 0.076). **c** Heatmap displaying the clustered genes in the leading edge subset of cardiovascular-related gene sets with their overlap with the GO terms. The shades of blue varies with the down regulation of the DEG in the absence of HIRA (Darker blue is for the most downregulated DEG). **d** qRT-PCR validation and RNAseq results displayed by fold changes in *Hira*-null compared to WT differentiated cardiomyocytes of a selection of DEG involved in heart development. Two different biological replicates were used for RNA-seq. Technical replicates from a single experiment for the q-PCR is displayed and is representative of three different biological replicates. **e** KEGG pathway analysis using GSEA on the DEGs in the absence of HIRA (adjusted *p* value ≤ 0.05 and FC = ± 2). X-axis shows the normalized enrichment score (NES) with the bars on the right and left sides of the Y-axis showing pathways that are positively and negatively enriched respectively. (Color figure online)

Gene Set Enrichment Analysis (GSEA) was performed on the DEGs and we identified 52 gene sets negatively enriched and 54 gene sets positively enriched in the absence of HIRA (*p* value ≤ 0.01 and FDR ≤ 0.1). Enrichment map was used to visualize and functionally categorize these different gene sets, and showed an under representation of GO-terms related to cardiovascular development, immune system development and DNA repair, and over representation of GO terms related to nervous system development and neurotransmission (Fig. 2a). Accordingly, the gene sets related to cardiac septum development and cardiac chamber development were both negatively enriched (Fig. 2b). We then examined the genes that were common in the different cardiovascular development gene sets using the lead edge analysis tool in GSEA. We found that *Gata4, Gata6, Hand1, Hand2, Isl1 and Tbx2*, and other genes represented in Fig. 2c, were common in several gene sets. We then validated a subset of these genes by qRT-PCR from independently isolated mRNA sample sets (Fig. 2d).

Altogether, the expression of cardiovascular development-related genes, and more precisely cardiac chamber and septal development associated genes was down regulated in the absence of HIRA.

### HIRA affects calcium signalling and vascular smooth muscle contraction pathways

We then investigated the pathways affected by the absence of HIRA. KEGG pathway analysis was done on the pre-ranked list of DEGs using GSEA (*p* value ≤ 0.05 and FC = ± 2). Interestingly, calcium signalling pathway was the most positively enriched (normalized enrichment score (NES) = 1.82, *p* value = 0.019 and FDR = 0.19). In addition, axon guidance and neuroactive ligand-receptor interaction pathways, two pathways related to the nervous system, were positively enriched. Conversely, the vascular smooth muscle contraction pathway was negatively enriched (NES = − 1.63, *p* value = 0.026 and FDR = 0.17). Considering the studies that link HIRA to interferon-stimulated genes [34], it is interesting to mention that leukocyte transendothelial migration pathways, which is related to the immune system is also negatively enriched (Fig. 2e).

### HIRA-dependent H3.3 deposition co-localizes with active enhancer loci in WT cells and shifts to distal intergenic regions in the absence of HIRA

We previously showed that H3.3 deposition at the cardiac enhancer, *Tte*, is HIRA dependent [20]. However, there has been no study investigating the genome-wide effect of HIRA on the H3.3 deposition during the early stages of cardiovascular development. We therefore performed H3.3-HA ChIP-seq on undifferentiated mESCs and differentiated cardiomyocytes at day 15 that contained about 60% of beating foci and 67% of transcriptome similarly to the previously published CM stage [7].

**Fig. 3:**
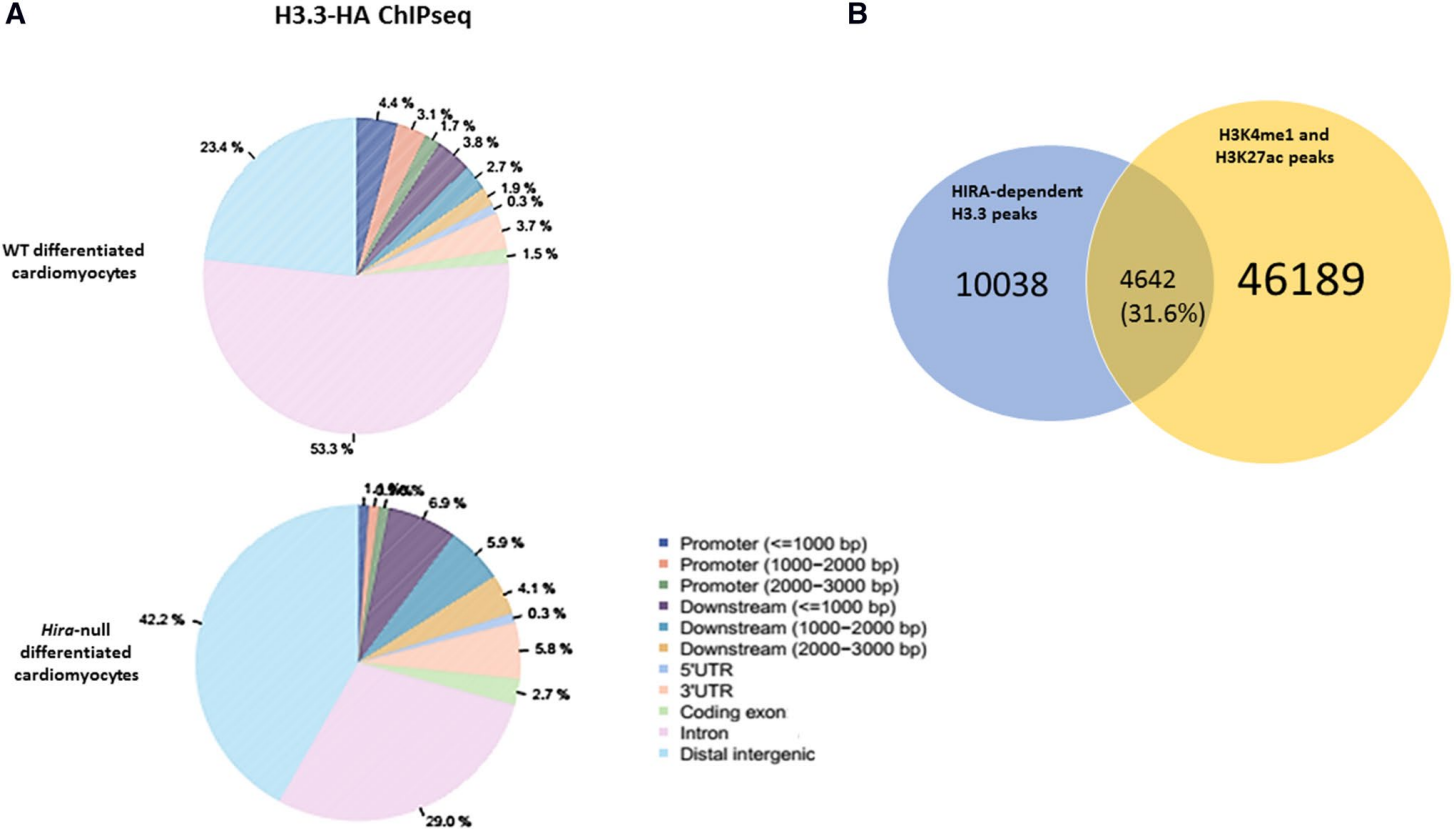
Genome wide distribution of H3.3 in WT and *Hira*-null differentiated cardiomyocytes and motif analysis of the significant peaks. **a** Pie chart showing the distribution of H3.3 over different genomic regions in WT and *Hira*-null differentiated cardiomyocytes as indicated. **b** Venn diagram showing the overlap between HIRA-dependent H3.3 peaks and the common peaks of H3K4me1 and H3K27ac. (Color figure online)

In WT-differentiated cardiomyocytes, H3.3 was deposited most abundantly in the intronic (53.3%) region with lesser deposition in the distal intergenic region (23.4%), and the promotor region (9.2%). In the absence of HIRA, the proportion of H3.3 deposition increased at the distal intergenic region (42.2%) compared to the intronic region (29%) and the promotor region (3%) (Fig. 3a). We identified 14680 HIRA-dependent H3.3 enrichment peaks encompassing +10 kb upstream of the TSS, the gene body or 200 bp downstream of the TES of 4703 annotated genes. GO analysis showed enrichment of DNA-dependent regulation of transcription processes. We then compared these with the DEG list and found 319 in common.

Interestingly, 60 of the genes enriched for HIRA-dependent H3.3 deposition were related to heart development by GO analysis. We then compared our datasets with previously published datasets for the known enhancer marks H3K4me1 and H3K27ac in cardiomyocyte-differentiated mESCs [7]. We identified that 31.6% (4642/14,680) of the total HIRA-dependent H3.3 enrichment peaks overlapped with both the active enhancer histone marks H3K4me1 and H3K27ac implicating that approximately one-third of the HIRA-dependent H3.3 deposition is at active enhancer sites (Fig. 3b).

In conclusion, the absence of HIRA leads to a decrease in the deposition of H3.3 in the intronic and promoter regions. We also show that about third of HIRA-dependent H3.3 peaks co localize with active enhancers loci.

### HIRA-dependent H3.3 deposition directly influences the transcription *of Meis1, Gata6* and *Tbx2*

We next investigated the co-localization of HIRA-dependent H3.3 deposition with some previously published ChIPseq datasets of cardiac transcription factors (NKX2.5, GATA4, TBX5 [35] and SRF [36]). We found that 1607 of the HIRA-dependent H3.3 peaks (10.93%) overlapped with NKX2.5 peaks, 1420 peaks (9.67%) overlapped with SRF peaks, 937 peaks (6.38%) overlapped with GATA4 peaks and 450 peaks (3.06%) overlapped with TBX5 peaks. Since we previously showed that HIRA and NKX2.5 bind to the common enhancer *Tte* [20], we focussed on identifying the colocalization of HIRA dependent H3.3 deposition and NKX2.5 at the enhancer loci and/or at the promotor site of three cardiac transcription factors *Meis1, Gata6* and *Tbx2*, which were downregulated in the absence of HIRA (Fig. 2d). These are known to be associated with cardiac septal defects, as we observed in our cardiac mesoderm *Hira*-conditional mice.

**Fig. 4:**
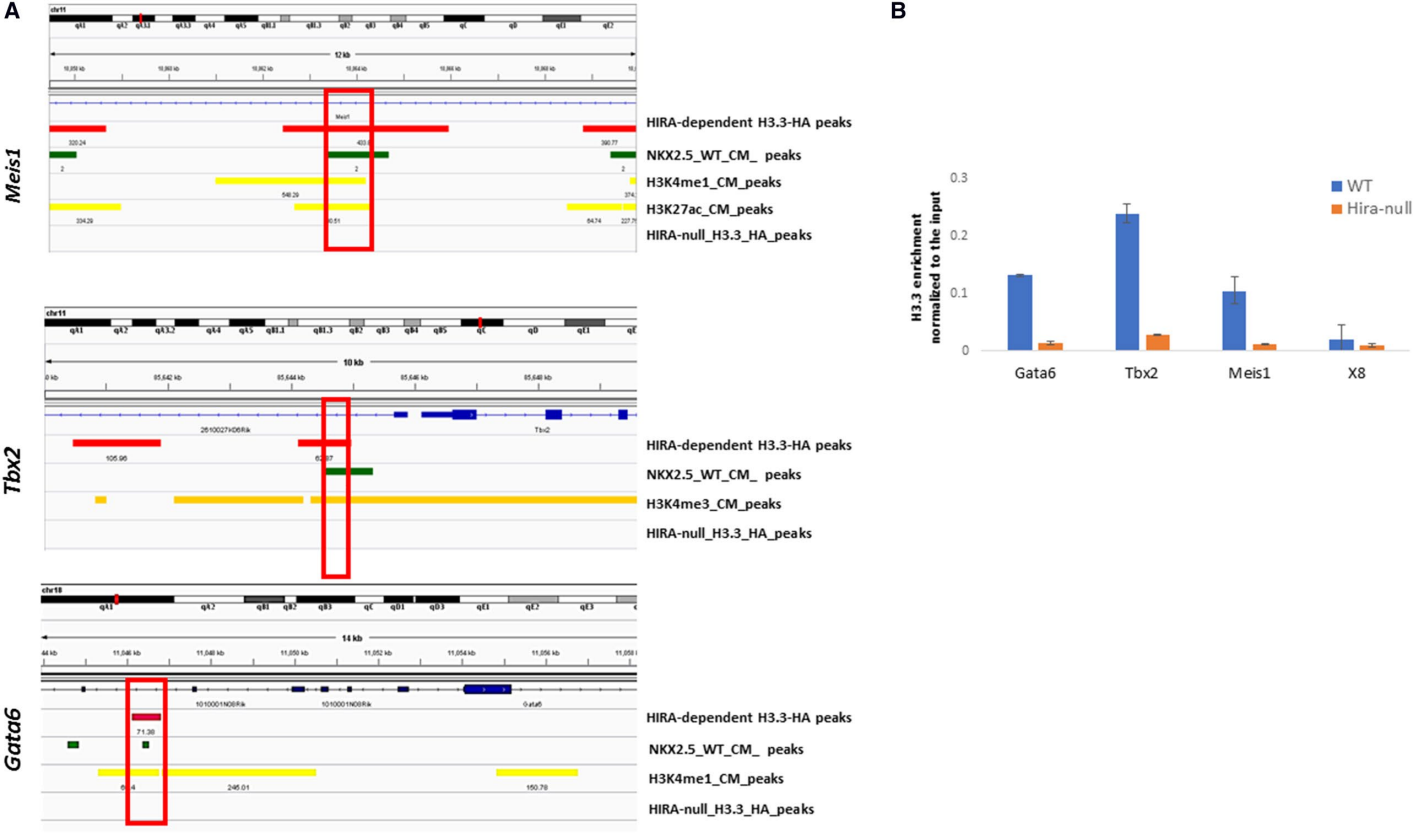
HIRA directly binds the enhancers of *Meis1* and *Gata6*, and the promotor of *Tbx2*, also occupied by NKX2.5. **a** IGV profiles of the different ChIP-seq peaks identified by MACS as indicated at the selected genes. **b** quantitative real time PCR following H3.3 ChIP at the indicated loci in WT and *Hira*-null differentiated cardiomyocytes at day15, the intergenic region in chromosome 8 (X8) was used as a negative control. Data were normalized to the input (= 1). (Color figure online)

MEIS1 is a homeobox protein found to influence heart development. Mouse embryos lacking MEIS1 showed VSDs and overriding of aorta [37]. We found several HIRA-dependent H3.3 enrichment sites in the gene body of *Meis1* (Fig. 4a). One of the most H3.3-enriched loci co-localizes with NKX2.5 at an enhancer locus marked by H3K4me1 and H3K27ac. We validated this enrichment by ChIP followed by qPCR using primers designed for this locus.

TBX2 is a member of the T-box transcription factor gene family. Absence of TBX2 leads to outflow tract septation defects [4]. We found HIRA dependent H3.3 enrichment in the promoter region, 1.1 kb from the TSS. This enrichment peak overlaps with H3K4me3 enrichment [7] and NKX2.5 (Fig. 4a). We validated this result by ChIP followed by qPCR using primers designed specifically for this locus (Fig. 4b).

GATA6 is a zinc finger transcription factor that plays a role in heart development. Lack of GATA6 has been associated with septal and outflow tract defects [38]. HIRA-dependent H3.3 enrichment was detected at approximately 5.5 kb from the TSS (Fig. 4a). This enrichment, that we validated by ChIP followed by qPCR (Fig. 4b), co-localizes with NKX2.5 and H3K4me1 suggesting an active role of HIRA in the transcription of *Gata6*. Interestingly, this locus has been reported previously to be an enhancer for *Gata6* [39].

Altogether, HIRA-dependent H3.3 is enriched at previously identified enhancer loci, essential for the expression of the cardiac transcription factors MEIS1, TBX2 and GATA6 at the early cardiomyocyte stage.

## Discussion

Heart development is a complex tightly regulated process requiring strict control of temporal gene expression [40–42] and involving epigenetic regulation [43]. We examined the role of the H3.3 histone chaperone HIRA in the early stages of heart development using an in vitro mESC differentiation model. We successfully differentiated both WT and *Hira*-null mESCs into cardiomyocytes. This was evidenced by the enrichment of heart development and morphogenesis GO terms.

We report new direct targets of HIRA-mediated H3.3 deposition, which are transcription factors known to be involved in cardiac septum formation. We previously demonstrated in vivo that mesodermal conditional *Hira*-null embryos presented with a fully penetrant phenotype of ventricular septal defect [20]. In that same study, we focussed on the dysregulation of expression of *Tnni2* and *Tnnt3*, suggesting a mechanism in which HIRA with NKX2.5 binds to their common enhancer *Tte* to down regulate their expression. We herein predict further targets of the same mechanism: co-occupancy of HIRA and NKX2.5 at the enhancers of *Meis1* and *Gata6*, and at the promoter of *Tbx2* to activate their expression. Further studies will be needed to investigate the downstream effect of HIRA on these new targets in vivo, and to explore the interactions of HIRA and NKX2.5 at these loci.

Interestingly, knockout of *Meis1* is associated with VSD and overriding aorta in mice [37], and an increased cardiomyocyte proliferation in zebrafish, suggesting a role in the cell cycle [44]. In our differentiated cardiomyocytes, *Meis1* was down regulated (FC = − 2.5) in the absence of HIRA. Notably, there was co-localization of HIRA-dependent H3.3 deposition with NKX2.5 binding sites at a previously identified enhancer site. A previous study showed that the sequential binding of MEIS1 then NKX2.5 to enhancers can influence cardiogenesis [45]. Taken together with our previously published finding, which showed diminished binding of NKX2.5 at the enhancer site *Tte* in the absence of HIRA, we hypothesize that the absence of HIRA leads to a diminished expression of *Meis1* and then to a diminished binding of NKX2.5 to the MEIS1-NKX2.5 target sites, thereby disrupting cardiac septum morphogenesis.

Similarly, *Gata6* expression was down regulated (FC = − 6.1) in the absence of HIRA at day 15 of differentiation, and we identified HIRA-dependent H3.3, H3K4me1 and NKX2.5 enrichments co-localising at 5.5 kb upstream of the TSS of *Gata6* in WT cells at day 15 of differentiation. It is interesting to note that the identical locus had been shown previously to be an enhancer bound by NKX2.5 and regulating *Gata6* expression in developing murine hearts [39].

TBX2 plays an essential role in the atrioventricular canal and OFT development [4]. We found HIRA-dependent H3.3 deposition at the promotor of *Tbx2* in WT cells differentiated at day 15 and a down regulation of *Tbx2* expression (FC = − 4.6) in the absence of HIRA at day 15 of differentiation. This locus coincides with known NKX2.5 binding sites and a peak of the active transcription mark H3K4me3. This result suggests that HIRA may play a role in the atrioventricular canal and the OFT development by influencing the transcription of *Tbx2* through H3.3 deposition at its promotor.

In addition, we found that calcium signalling pathway was significantly upregulated in the absence of HIRA at day 15 of differentiation. Calcium signalling controls several physiological processes in the heart, including cardiac contractility [46]. KEGG calcium signalling pathway is normally required in ESC-derived cardiomyocytes [47]. We previously reported two cardiac genes involved in contractibility to be up regulated in the hearts of cardiac mesoderm *Hira*-conditional mice. Further investigations on cardiac contractility in *Hira*-mutant mice would be interesting.

Outside of heart development, it is of interest to note that in our study, gene sets involved in DNA repair, the development of the immune and nervous systems were significantly dysregulated in the absence of HIRA. Of relevance, HIRA has been shown to have an essential role in transcriptional recovery after DNA damage [48, 49], and several studies link HIRA-dependent H3.3 deposition with the induction of stress responsive associated genes following interferons and heat-shock stimulations [34, 50]. Absence of HIRA in neural progenitors resulted in decreased proliferation and increased neuronal differentiation [51]. Our study provides new subsets of genes that could be investigated further in these areas.

In conclusion, we show that cardiovascular development-related genes are expressed in both WT and *Hira*-null differentiated cardiomyocytes with a significant lower expression in *Hira*-null cells. We show that 31.6% of HIRA-dependent H3.3 peaks co-localize with active enhancers loci. We found that HIRA directly influence the expression of the cardiac transcription factors *Meis1, Gata6* and *Tbx2*. We suggest that HIRA influences their expression through the deposition of H3.3 at their enhancers, supporting our previous published model that HIRA acts at enhancers during cardiovascular development, and potentially in concert with NKX2.5 [20].
